# Toll-like receptor-mediated innate immunity orchestrates adaptive immune responses in HBV infection

**DOI:** 10.3389/fimmu.2022.965018

**Published:** 2022-07-29

**Authors:** Yanqin Du, Jun Wu, Jia Liu, Xin Zheng, Dongliang Yang, Mengji Lu

**Affiliations:** ^1^ Department of Infectious Diseases, Union Hospital, Tongji Medical College, Huazhong University of Science and Technology, Wuhan, China; ^2^ Institute for Virology, University Hospital Essen, University of Duisburg-Essen, Essen, Germany

**Keywords:** Toll-like receptor, hepatitis B virus, adaptive immunity, innate immunity, T cell response

## Abstract

Chronic hepatitis B virus (HBV) infection remains to be a substantial global burden, especially for end-stage liver diseases. It is well accepted that HBV-specific T and B cells are essential for controlling HBV infection. Toll-like receptors (TLRs) represent one of the major first-line antiviral defenses through intracellular signaling pathways that induce antiviral inflammatory cytokines and interferons, thereby shaping adaptive immunity. However, HBV has evolved strategies to counter TLR responses by suppressing the expression of TLRs and blocking the downstream signaling pathways, thus limiting HBV-specific adaptive immunity and facilitating viral persistence. Recent studies have stated that stimulation of the TLR signaling pathway by different TLR agonists strengthens host innate immune responses and results in suppression of HBV replication. In this review, we will discuss how TLR-mediated responses shape HBV-specific adaptive immunity as demonstrated in different experimental models. This information may provide important insight for HBV functional cure based on TLR agonists as immunomodulators.

## 1 Introduction

Hepatitis B virus (HBV) infection affects approximately 3.5% of the world’s population and remains a major cause of end-stage liver disease, such as cirrhosis and hepatocellular carcinoma (HCC) (1). HBV-specific adaptive immune responses play an essential role in HBV clearance. During acute HBV infections, vigorous HBV-specific T-cell responses contribute to viral clearance (2). However, HBV-specific T cells exhibit quantitative and functional defects accompanied by an exhausted phenotype with upregulation of several coinhibitory molecules during chronic HBV infection (3). This is considered as a major contributing factor to HBV persistence. Currently available treatment regimens, including pegylated interferon alpha (Peg-IFN-α) and nucleos(t)ide analogs (NAs), can effectively suppress HBV replication but rarely achieve a functional cure. In recent years, various immunotherapeutic agents that aim to restore HBV-specific immune responses have been investigated, including anti-PD1 antibodies (4), CTLA4 inhibitors and pattern recognition receptor (PRR) agonists (5). However, these novel treatment options are still under preclinical or early clinical evaluation.

Toll-like receptors (TLRs), members of the evolutionarily ancient family of PRRs, play a central role in responses to microbial pathogens by recognizing pathogen-associated molecular patterns (PAMPs) (6). Twelve and 10 functional TLRs have been identified in mice and humans, respectively (7). Based on the cellular localization and the respective PAMP ligands, TLRs are largely divided into two groups. TLR3, TLR7, TLR8, and TLR9 are localized in intracellular vesicles, such as endosomes, endoplasmic reticulum (ER), and lysosomes, and recognize viral DNA (TLR9) or RNA [double-stranded RNA (TLR3), single-stranded RNA (TLR7 and TLR8)] (8, 9). TLR1, TLR2, TLR4, TLR5, and TLR6 are localized in the cell surface and recognize extracellular bacterial and fungal cell wall components and some viral proteins, including lipoproteins (recognized by TLR1, TLR2, and TLR6), lipopolysaccharide (LPS) (TLR4), and flagellin (TLR5) (8). Upon recognizing respective PAMPs, TLRs selectively recruit distinct adaptor molecules, such as TRIF and MyD88, and initiate downstream signaling events that result in the secretion of type I interferon (IFN), inflammatory cytokines, and chemokines (9, 10). In this review, we will discuss the interaction between TLRs and HBV and how different TLR ligands regulate HBV-specific T-cell responses.

## 2 TLR-mediated innate responses to HBV infection

### 2.1 Recognition of HBV by TLRs

The recognition of HBV by the innate immune system involves three types of host cells: hepatocytes, innate immune cells, such as dendritic cells (DCs) and macrophages, and hepatic non-parenchymal cells (11). Previously, acute HBV infection has been reported to only weakly induce the expression of type I IFN and innate immune genes within the liver of infected animals (12) and patients (13–15). However, Hösel et al. found that HBV could be recognized by Kupffer cells (KCs), present in primary human hepatocytes (PHHs) culture *in vitro*. This recognition leads to the activation of the NF-κB signaling pathway and the subsequent release of proinflammatory cytokines but does not induce an interferon response in KCs (16). Moreover, a recent study also showed that infection of PHHs with HBV induces the secretion of proinflammatory cytokines through TLR2 signaling but not IFNs (17). Consistent with these findings, another study showed that mouse B cells could be activated by HBV particles through the TLR2–MyD88–mTOR axis (18). Furthermore, HBsAg has been reported to be recognized by TLR4 on monocytes or myeloid DCs *via* CD14 and increase the production of IL-10 (19, 20). These data suggest that host innate immunity could indeed sense HBV infection, although it may be weak.

Interestingly, the genetic single-nucleotide polymorphisms (SNPs) of TLRs have potential effects on the outcome of HBV infection. The TLR3 (rs3775290, rs3775291) and TLR4 (rs4986790) SNP variants link to a higher risk of chronic HBV infection and HCC (21, 22), while mutations of rs3804099 and rs4696480 in TLRs correlate with HBsAg reduction and liver function improvement (23).

### 2.2 Impaired expression and function of TLRs in HBV infection

During chronic infection, HBV modulates TLR response (24). The impaired expression of TLRs in immune cells from patients with chronic hepatitis B infection (CHB) has been reported in several studies. Peripheral blood mononuclear cells (PBMCs) from CHB patients displayed a significantly reduced expression of TLR transcripts, including TLR1, 2, 4, and 6 (25). Similarly, PBMCs from CHB patients showed a reduced expression of TLR3 (26), TLR8 (27), TLR7, and TLR9 (28, 29) as well as the TLR signaling molecules IRAK4, TRAF3, and IRF7 (30). Additionally, patients who achieved a complete response sustained higher TLR8 mRNA levels in PBMCs than non-responders at week 12 after Peg-IFN-α therapy (27). Consistent with this finding, partial restoration of TLR2 and TLR3 expression in PBMCs has been observed in patients with virological response after treatment (26, 31).

In addition to downregulating TLR expression, HBV infection also impairs the functional response of TLR signaling. PBMCs from CHB patients exhibit impaired cytokine secretion after challenging with TLR2, TLR4 (25, 32), and TLR 8 ligand (27). Consistent with this finding, PBMCs and plasmacytoid DCs (pDCs) from CHB patients have shown significantly decreased IFN-α production in response to TLR7 and TLR9 ligands (33, 34). Indeed, HBV components have been reported to interrupt the intracellular signaling pathways of TLRs. HBsAg inhibits IRF7 expression and nuclear translocation in pDCs (34) and also interferes with the NF-κB pathway by interacting with the TAK1–TAB2 complex (35). Moreover, HBeAg has been reported to disrupt homotypic TIR : TIR interactions and thus suppress TIR-mediated activation of the NF-κB and IFN-α promoters (36). Furthermore, HBV polymerase inhibits the activity of IKKs and thereby suppresses TLR3- and TLR4-induced NF-κB signaling (37). Wu et al. showed that supernatant from TLR3-stimulated liver sinusoidal endothelial cells (LSECs) suppressed HBV replication in hepatocytes (38). However, HBV components, such as HBsAg, HBeAg, and HBV virions, suppressed the activation of IRF-3, NF-κB, MAPK, and ERK 1/2 and abrogated TLR-induced antiviral activity in LSECs (39, 40). Taken together, these studies suggest that various HBV components can interrupt the TLR signaling pathway, which might explain the impaired function of the TLR signaling pathway during HBV infection.

Of note, persistent inflammation in CHB patients is also partly responsible for the impairment of TLR response. For instance, IFN-α production by pDCs in response to TLR7 or 9 ligands is negatively correlated with alanine aminotransferase (ALT) levels (41, 42).

## 3 Regulation of HBV-specific immune responses by TLR agonists

TLR agonists may directly inhibit HBV replication in hepatocytes or indirectly suppress HBV replication by antiviral cytokines produced by other innate immune cells (43, 44). On the other hand, HBV-specific T- and B-cell responses ultimately determine the outcomes of HBV infections. In chronic HBV infections, HBV-specific T cells display quantitative and functional defects, a state referred to as T-cell exhaustion (45). Moreover, exhausted T cells are more susceptible to tumor necrosis factor-alpha (TNF-α)-related apoptosis-inducing ligand (TRAIL)-dependent NK-cell-mediated lysis due to the upregulation of the TRAIL death receptor (46). Therefore, restoring HBV-specific T-cell responses in CHB patients represents a promising strategy to achieve HBV functional cure.

**Figure 1:**
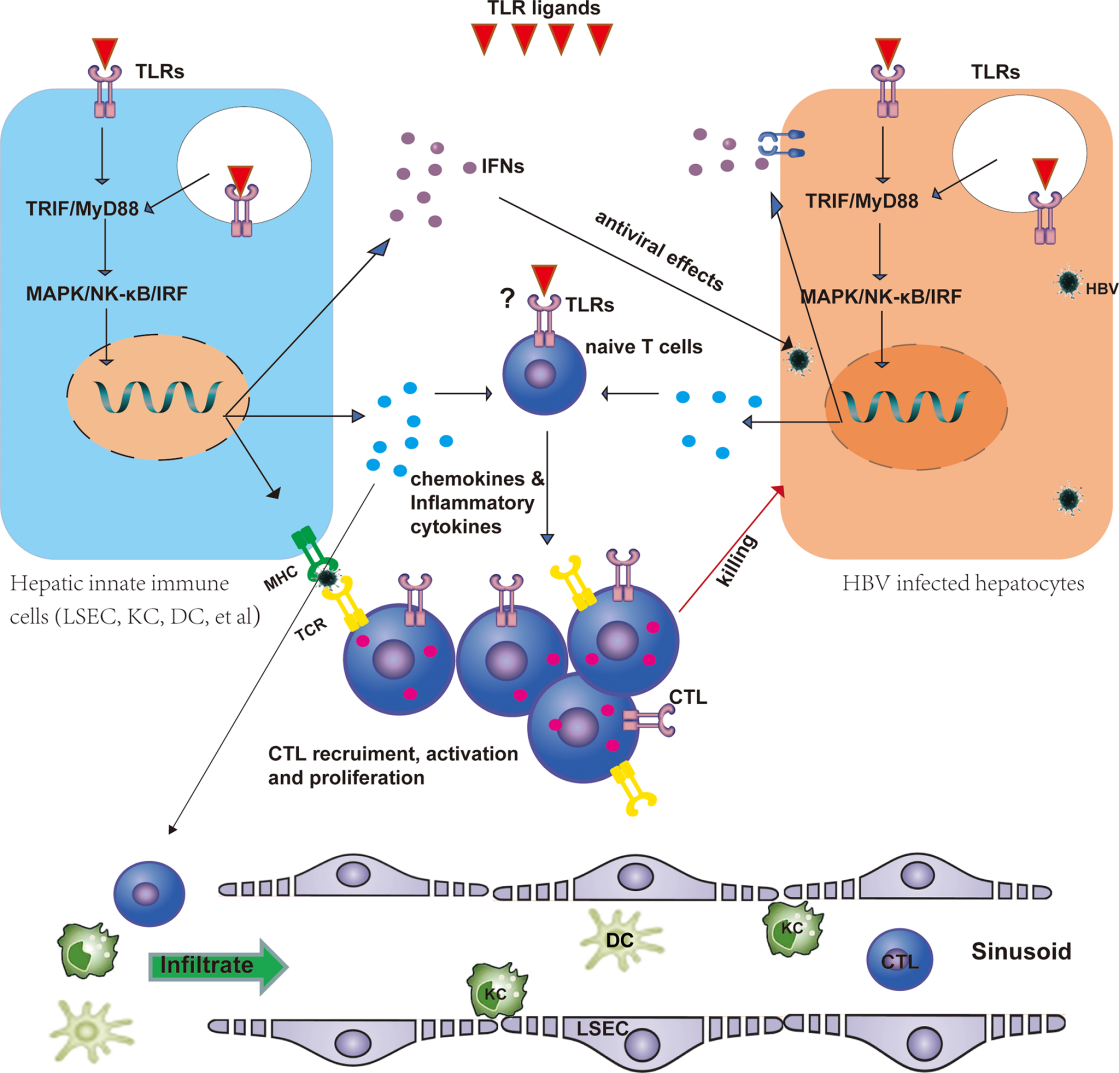
TLRs regulate HBV-specific T-cell responses. TLRs are expressed in T cells, hepatocytes, and hepatic non-parenchymal cells, including LSECs, KCs, and DCs. TLRs may directly shape the T-cell response as costimulatory molecules. Stimulation of TLRs by their ligands leads to the activation of the downstream MyD88/TRIF-dependent signaling pathway in hepatic non-parenchymal cells and promotes the maturation of these cells, thus promoting antigen presentation to T cells and the production of IFNs, proinflammatory cytokines, and chemokines. IFNs exert antiviral effects against HBV in infected hepatocytes. Chemokines and inflammatory cytokines recruit DCs, macrophages, and specific T cells into the liver and promote HBV-specific T-cell activation and proliferation. Activated CLTs then kill infected hepatocytes. CTL, cytotoxic T cell; DC, dendritic cell; HBV, hepatitis B virus; IFN, interferon; LSEC, liver sinusoidal endothelial cell; KC, Kupffer cell; TLR, Toll-like receptor.

Several studies have investigated the effects of TLR agonists to restore HBV-specific CD8^+^ T-cell responses. The liver is a specialized immunological organ with a unique composition of innate immune cells, including liver resident cells, such as parenchymal cells, and non-parenchymal cells, that is, LSECs, KCs, and hepatic stellate cells, and recruited immune cells, such as DCs, macrophages, and T and B cells. Activation of TLR signaling in these hepatic cells can induce the production of type I IFN and a variety of proinflammatory cytokines, such as TNF, IL-6, IL-12, and IL-18 (47), which play essential roles in controlling HBV replication (38, 48) but also modulate specific immune responses (**Figure 1**). For instance, IL-6 could control the expression of HBx and suppress HBV replication through modulating the activity of HBV enhancer I, and it also participates in the activation of NK cells and cytotoxic T cells (CTLs) (49). IL-12 and IL-18 could rescue the exhausted CD8 T-cell responses (50) or promote the secretion of IFN-γ by T cells (51).

### 3.1 TLRs directly shape the T-cell response

Recent studies have stated that TLRs may serve as costimulatory molecules on T cells (52, 53). The expression of TLR2 is detected in activated CD8 T cells on their surface, and TLR2 stimulation reduces the requirement of costimulatory signals delivered by antigen-presenting cells (APCs) and directly promotes their differentiation, proliferation, and effector function (53–55). A recent study also demonstrated that the TLR2 agonist P3C could directly enhance TCR-dependent CD8 T-cell activation by increasing cellular glycolysis and glutaminolysis (52). Similarly, the TLR7 ligand also enhanced the effector functions of TCR-dependent CD8 T cells primed by CD3, and the effects were accompanied by upregulation of glucose uptake and glycolysis (56). Moreover, human effector CD8^+^ T cells constitutively express TLR3 and TLR9. Stimulation of these TLRs by respective ligands directly promotes IFN-γ production by T cells (57, 58). Overall, these data suggest that activation of TLR signaling in T cells directly promotes T-cell activation, proliferation, and effector functions. However, the direct effect of TLR ligands on T cells in the treatment of CHB requires further investigation.

### 3.2 TLRs indirectly shape the adaptive immune response through innate immune cells

#### 3.2.1 TLRs regulate T-cell immunity through DCs

As professional APCs, DCs exhibit a potent capacity to prime naïve T cells and induce them to develop into different subtypes of T cells based on the origin of the maturation signals. There are two major DC subsets identified in humans: myeloid DCs (mDCs) and pDCs (59). These cells express different sets of TLRs. Specifically, pDCs predominantly express TLR7 and TLR9, while mDCs express a wider range of TLRs, including TLR1, TLR2, TLR3, TLR4, TLR5, TLR6, and TLR8 (60). Therefore, pDCs can sense the nucleic acids of viral pathogens through TLR7 and TLR9 and induce the secretion of type I IFN. pDCs exhibit a dysfunctional phenotype during chronic HBV infection, indicated by downregulation of TLR9 (29, 61) and reduction in antigen-presenting and migration capacity (62) and decreased production of IFN-α and inflammatory cytokines in the response to TLR7 and 9 ligands (29, 33, 34, 41, 63). The functional deficit of DCs might participate in T-cell dysfunction given the impaired interaction between DCs and T cells, including failure of DCs to increase HLA-II and costimulatory molecules and defective induction of antigen-specific CLT proliferation and cytokine production (64). However, two studies stated that the ability of pDCs in PBMCs to stimulate T-cell proliferation was similar between CHB patients and healthy control (42, 65). Despite these discrepancies, therapeutic vaccines targeting DC function might represent an opportunity to improve HBV-specific T-cell responses.

Some studies have investigated TLR agonists as vaccine adjuvants to improve DC function and thus induce HBV-specific T-cell responses. Synthetic long peptides (SLPs) are linear amino acid sequences that are most efficiently presented by DCs (66). Dou et al. found that the presence of the TLR2 ligand promoted the effects of HBV core protein-derived SLP to boost CD4 and CD8^+^ T-cell responses in CHB patients *ex vivo* (67). Later, the same group identified that HBV-core SLP that conjugates with the TLR2 ligand also triggered a functional T-cell response, but it reduced the cross-presentation efficiency of the SLP-containing epitope by DC subsets (68). However, the cross-presentation can be improved by either placing a valine–citrulline linker between the TLR2 ligand and the long SLP or by shortening the SLP (68).

#### 3.2.2 TLRs regulate T-cell immunity through hepatocytes or non-parenchymal liver cells

Murata et al. demonstrated that antigen presentation by hepatocytes is more efficient than by hematopoietic cells for inducing HBV-specific CD8^+^ T-cell responses in the liver (69). Yan et al. reported that the TLR5 ligand flagellin (SF) could modulate the intrahepatic CD8^+^ T-cell response by regulating the responses of hepatocytes (70). Coculture of SF-treated primary mouse hepatocytes with splenocytes results in the activation of CD8^+^ T cells in the coculture system during anti-CD3 stimulation or antigen-specific activation (70).

LSECs are liver-resident APCs that have competent capacity in antigen cross-presentation to CD8^+^ T cells (71). However, the antigen-specific interaction of LSECs with CD8^+^ T cells does not induce T-cell activation under physiological conditions (72). Nevertheless, T-cell tolerance induced by LSECs can be regulated by several factors, such as NOD1 ligand, DAP (73), TLR2 agonist (74), and combinatorial stimulation of CD28 and IL-12 (75). Liu et al. illustrated that stimulation of LSECs with the TLR1/2 ligand promoted the maturation of LSECs and enabled them to further activate virus-specific CD8^+^ T cells in mice (74). Mechanistically, IL-12 produced by LSECs was an essential mediator of LSEC-mediated CD8^+^ T-cell immunity (74).

KCs are resident macrophages in the sinusoids of the liver that express TLRs and secrete large amounts of inflammatory mediators that regulate antiviral immunity during HBV infection (76). Human KCs express TLR2–4, whereas KCs from rats and mice express TLR1-9 (26). Previous data demonstrated that the TLR2 ligand P3C further strengthens the tolerogenic and suppressive properties of intrahepatic myeloid-derived cells (iMDCs) in mice. Mechanistically, the enhanced inhibition of T-cell activation was mainly induced by KCs *via* secreting IL-10 (77). Consistent with this finding, another group identified that KCs support HBV-mediated CD8 T-cell exhaustion *via* the HBV core antigen–TLR2 interaction in mice (78). On the other hand, KCs produce CXCL8 upon HBV exposure (16), which potentially attracts NK and NKT cells into the liver during the early phase of HBV infection. Uwatoku et al. found that KCs are crucial for DC recruitment to the liver through N-acetyl galactosamine-specific C-type lectin interactions (79). The increased DC recruitment may promote HBV antigen presentation and thus enhance HBV-specific T-cell responses. As antigen-presenting cells, mouse KCs also present antigens to T cells, thus inducing T-cell proliferation and the production of IFN-γ (80, 81). However, the antigen-presenting function of KCs is much weaker than that of DCs. A recent study identified a subset of KCs (KC2) that could cross-present hepatocellular antigens to CD8 T cells upon IL-2 administration and thus improve the antiviral functions of T cells (82).

### 3.3 TLR agonists regulate HBV-specific T-cell immunity by modifying the intrahepatic immune microenvironment and recruiting immune cells into the liver

In a persistent HBV replication mouse model utilizing hydrodynamic injection (HI) of the pAAV/HBV 1.2 plasmid, HBV-specific immune responses are primed but do not clear HBV from the liver (83). Therefore, this model was used to test the effects of TLR agonists on HBV-specific immune responses. Previously, our group indicated that intrahepatic application of TLR3 ligand poly(I:C) after establishment of persistent HBV replication efficiently recruited CD8^+^ T cells into the liver, enhanced HBV-specific T-cell responses, and cleared HBV in an IFN-γ- and CXCR3-dependent manner (84, 85). Later, we constructed calcium phosphate nanoparticles carrying poly(I:C) conjugated with F4/80, which promoted liver targeting by conventional intravenous injection. These nanoparticles exerted a similar enhancing effect on HBV-specific T-cell responses like HI of poly(I:C) (86). However, simultaneous or prior activation of TLR3 signaling by HI of poly(I:C) results in expansion of Tregs, KCs, and myeloid-derived suppressor cells (MDSCs), all of which impair the HBV-specific T-cell response and thus inhibit HBV clearance (85). Consistent with this finding, another study investigated the HBV-specific T-cell immunity and anti-HBV effect by TLR2 activation (P3C) in the same model at different time points, which found that only TLR2 pre-activation could enhance the intrahepatic HBV-specific T-cell response (87). In detail, pre-activation of TLR2 reduced the number of hepatic F4/80^+^ macrophages but increased the number of CD11c^+^ DCs, which is helpful for the initiation of the HBV-specific T-cell responses in the following time period (76). Huang et al. reported that TLR signaling induced intrahepatic aggregates of myeloid cells that enabled the population expansion of CTLs during chronic viral liver infection (88). Hepatic CTL proliferation was restricted to myeloid-cell aggregates for T-cell population expansion (iMATES) that were composed of inflammatory monocyte-derived CD11b^+^ cells (88). These findings suggest that the application of TLR ligands at the right time and in the right location can enhance virus-specific T-cell responses by recruiting immune cells into the liver. Of note, the HI mouse model is quite different from human beings. Therefore, the findings in mouse studies should be carefully evaluated and verified in human research.

## 4 TLR agonists in clinical research

TLR agonists are potential antiviral agents in chronic HBV infection. Indeed, several studies have investigated the anti-HBV effects of TLR ligands in clinical studies (**Table 1**). An earlier study tested the efficacy of polyadenylic.polyuridylic acid [poly(A).poly (U), TLR3 ligand] in CHB patients and found that normalization of ALT levels and HBeAg seroconversion were noted in approximately 57.9% of treated patients, suggesting that poly(A).poly (U) may be effective in the treatment of CHB patients (89). The safety and efficacy of the TLR7 agonist GS9620 have been assessed in CHB patients (90–92). GS9620 promoted ISG-15 expression, HBV-specific T-cell responses, and NK-cell activation and function and reduced the ability of NK cells to lysis T cells. However, HBsAg levels are not significantly reduced in the treated patients (90, 91, 93). Another study used different TLR agonists to stimulate mononuclear cells derived from chronic HBV- or HCV-infected livers and found that the TLR8 ligand ssRNA40 could induce the production of IFN-γ in chronic HBV- or HCV-infected livers by mucosal-associated invariant T cells (MAIT) and NK cells (94). Media from PBMCs that were stimulated with the TLR8 agonist GS-9688 reduced HBV replication in HBV-infected PHHs (95). A recent study found that GS-9688 could activate antiviral effector function in PBMCs from CHB patients by multiple immune mediators (HBV-specific CD8^+^ T cells, CD4^+^ follicular helper T cells, NK cells, and MAIT) (96). Similarly, TLR8 agonists enhance HBV-specific B-cell responses *via* improving monocyte-mediated follicular helper T-cell function in CHB patients (97). Taken together, the agonists of TLR3, 7, and 8 may serve as potential antiviral agents against chronic HBV infections, but further investigation is needed to evaluate their toxicity, tolerated range, and efficacy when used alone or applied together with current antiviral drugs.

**Table 1:** TLR agonists in clinical studies.

Compounds	Target	Clinical phase	Patients chosen	Effects on ALT and HBV	Effects on host immunity	Ref
poly(A).poly(U)	TLR3 ligand	Phase I	HBeAg-positive chronic hepatitis	73.7% normalization of ALT, 57.9% HBeAg seroconversion, 63.1% loss of HBV-DNA	No data available	(89)
Vesatolimod (GS-9620)	TLR7 ligand	Phase II (number: GS-US-283-1059, NCT 02166047)	Virally suppressed patients	No significant decline of HBsAg	Dose-dependent induction of ISG15, increased T-cell and NK-cell responses, and reduced ability of NK cells to suppress T cells	(90, 93)
Vesatolimod (GS-9620)	TLR7 ligand	Phase II (number: NCT 02579382)	Patients who are not currently on antiviral treatment	No significant reduction in HBsAg	Dose-dependent induction of ISG15	(91)
Selgantolimod (GS-9688)	TLR8 ligand	Preclinical study	PBMCs from CHB patients	Reduction of viral markers in HBV-infected PHH treated with media from PBMCs stimulated with GS-9688	Induced cytokines that activate antiviral effector function	(95, 96)

## 5 Conclusion

Despite the availability of an effective prophylactic vaccine, HBV infection remains a major challenge worldwide. During chronic HBV infection, HBV suppresses the expression of TLRs and downstream cytokines through various HBV components and thus limits HBV-specific adaptive immunity and suppresses virus clearance. Thus, restoration of HBV-specific immune responses may be essential for sustained viral control. Accumulated studies suggest that TLR-mediated innate immune responses could enhance HBV-specific responses and thus suppress HBV replication and expression. To achieve a functional cure of CHB, a combined strategy with current antiviral treatment, activation of TLR-mediated immunity, and restoration of HBV adaptive immunity should be investigated in future studies in both animal models and clinical trials. It will be useful to understand the various underlying mechanisms how TLRs mediate immune activations and identify those contributing to HBV-specific immune control. It is also important to find a way to direct immune cells to the liver and to let those to exert antiviral functions.

## Author contributions

YD and ML conceptualized and drafted this review. JW, JL, XZ, and DY edited the review. All authors contributed to the article and approved the submitted version.

## Funding

This work was contributed by a scholarship from the Medical Faculty of University of Duisburg-Essen, a grant from Deutsche Forschungsgemeinschaft (RTG 1949/2), and grants of National Natural Science Foundation of China (81672022 and 82170636).

## Conflict of interest

The authors declare that the research was conducted in the absence of any commercial or financial relationships that could be construed as a potential conflict of interest.

## Publisher’s note

All claims expressed in this article are solely those of the authors and do not necessarily represent those of their affiliated organizations, or those of the publisher, the editors and the reviewers. Any product that may be evaluated in this article, or claim that may be made by its manufacturer, is not guaranteed or endorsed by the publisher.
